# Epidemiological Society

**Published:** 1897-01-23

**Authors:** 


### Medical Progress and Hospital Clinics.

[*The Editor will be glad to receive offers of co-operation and contributions from members of the profession. All letters should be addressed to* The Editor, at the Office, 28 & 29, Southampton Street, Strand, London, W.C.]

### EPIDEMIOLOGICAL SOCIETY.

At the meeting held last Friday Dr. W. H. Hamer read a paper on "Age-incidence in Relation with Cycles of Disease-prevalence." The argument was of a highly technical description, and would be very difficult to follow without the diagrams and figures by which it was illustrated. The point, however, which Dr. Hamer demonstrated was that when such diseases as measles, scarlet fever, and small-pox varied in prevalence, so far as was shown by the mortality returns, they also varied in their distribution among the different ages. Several reasons had been suggested to explain the cyclical variation in the prevalence of these diseases. On one hypothesis explanation of the recurrence of epidemics after certain periods was to be sought in the fact that "certain years of life are prone to the disease, and that when these have been cleared away the disease could not prevail extensively until there were again persons of fit age to receive the poison." Dr. Ransome, however, had showed that this simple "age" theory was not capable of explaining the phenomena. Another hypothesis was that there was a variation in the quality of the infection, or disease germ. This had been insisted on by Whitelegge, who, however, distinguished between what he called "super-added waves" (annual seasonal waves, milk epidemics, and water epidemics) and the broader cycles determined by altered quality of the contagion; the former being attended with a lowered case mortality and a less severe average attack, while in the latter the intensification of the quality of the disease was attended by greater severity of attack, greater power of overcoming comparative insusceptibility, and greater power of epidemic diffusion.

A third hypothesis was that a certain density of population at susceptible ages was necessary before a disease could spread with the vigour of an epidemic. The last hypothesis would appear to be but different statements of the first, the idea of density being added to that of number.

It appeared, however, to Dr. Hamer that none of these views could be definitely accepted until we knew what was the composition, as regards age, of the groups attacked in epidemic as compared with non-epidemic times. He had, therefore, worked out the incidence of the diseases in question, as shown by their mortality, upon the different ages given in the London returns for each year for many years past, and had prepared a series of diagrams showing the proportion borne by the number affected at each age in successive years to the number affected at all ages during those years. Clearly, if the proportion of the different ages attacked in different years remained the same, the "curve" for each age would be a straight line; but if in epidemics one age were attacked in greater proportion than usual, the "curve" for that age would rise in such epidemic years. As a fact, the curves did rise and fall; and, of course, whenever one went up another went down to make the balance true, and thus Dr. Hamer was able to prove, what perhaps one might have expected, that the age-composition of the mortality from these diseases varied considerably from year to year. They did not, however, vary quite as one might have expected, and here came in the interest of the paper.

Roughly, one may say, although Dr. Hamer did not thus express it, that if the "age" theory were true, we might reasonably expect that in an epidemic which had arisen in consequence of a number of susceptible children having come into existence, the mortality would be composed in larger proportion of young children than in ordinary times. This, however, did not prove to be the case. Taking scarlet fever Dr. Hamer showed that between 1859 and 1893 the chart for the age period, 0 to 1, showed several correspondences between a high rate of mortality at that age and a comparatively low rate of mortality at all ages. The converse was also true, viz., that a low rate of proportionate mortality at that age coincided with a high rate of mortality at all ages. In the age period 1 to 2 the indications were less marked, but still tended to the same effect. When, however, we came to the age periods 4 to 5, and 5 to 10, there were clear indications, particularly marked in the earlier years of the series, of a correspondence between high comparative mortality at those ages and high general mortality.

Dr. Hamer entered into many other questions raised by his tables. We content ourselves, however, with reporting his demonstration of this one fact, which is most curious and interesting, that although, as is well known, the greater part of the deaths from scarlet fever always occur in children under four years of age, the increase in the mortality from that disease in times of epidemics is proportionately greater in children above that age, and that among infants under two years old, fatal as are the attacks, the mortality, compared with that at all ages, is less during epidemics than at other times. Whether prevalence makes virulence, or virulence make prevalence is another matter.

